# A Policy Framework for Producing Age-Friendly Communities from the Perspective of Production of Space

**DOI:** 10.3390/ijerph19042031

**Published:** 2022-02-11

**Authors:** Jianbo Han, Edwin H. W. Chan, Esther H. K. Yung, Queena K. Qian, Patrick T. I. Lam

**Affiliations:** 1 Department of Building and Real Estate, The Hong Kong Polytechnic University, Hong Kong SAR, China; blacksunset1116@hotmail.com (J.H.); esther.yung@polyu.edu.hk (E.H.K.Y.); bsplam@gmail.com (P.T.I.L.); 2 School of Public Administration, Hunan University, Changsha 410082, China; 3 Faculty of Architecture and The Built Environment, Delft University of Technology, 2628 Delft, The Netherlands; k.qian@tudelft.nl

**Keywords:** age-friendly community, urban planning, the production of space, scientometric analysis, theory review, policy study

## Abstract

Given various hindrances in the macro context, how to efficiently develop age-friendly community policies requires further research. Currently, such kinds of frameworks are lacking. This paper aims to develop a policy framework to minimise cost and resolve conflict of interest between different generations in age-friendly community development. The study adopted a scientometric method to review the theoretical development of age-friendly community studies. Firstly, with a search for the keywords “age-friendly” and “community” on Web of Science, 72 English academic papers were found containing explicit theories. Most of the studies were conducted in the Global North. Then, a mixed analytical method was used to find a suitable theory, “the production of space”, to develop the policy framework. Lastly, a policy framework was developed to overcome barriers to age-friendly community development strategically. Echoing previous studies, this paper proposes a way to counter financial austerity in age-friendly initiative investment and balance the consideration for older and younger populations in urban development. For practice, the policy framework can provide a reference for more efficient age-friendly community policymaking in different regions. For future research, the framework provides a model for more empirical studies considering the social dynamics in age-friendly community development.

## 1. Introduction

Population ageing has become significant all over the world. According to the World Health Organization’s projection, in 2050, the population aged 60 and over will reach 2 billion [1]. By that time, one-sixth of the world population will be older adults [2]. Population ageing can cause various challenging economic effects, including labour shortages, shrinking gross domestic product (GDP), and social care burdens [3]. Thus, the global socioeconomic situation will worsen without effective intervention due to an almost doubled dependency ratio in 30 years.

With rapid urbanisation, cities will be the home of a large portion of the older population in the future [4]. Well-planned cities and communities can provide necessary facilities to improve older residents’ quality of life. Scholars have already proved that both built environment and social infrastructures play an essential role to satisfy older residents’ daily demands [5]. Thus, creating a proper urban and community environment for older dwellers is vital for cities’ liveability.

To guide the development of policies regarding older people, the World Health Organization coined the concept of *active ageing* [6]. In addition to health, the concept also advocates older people’s participation and security. Then, the World Health Organization launched the concept of *age-friendly* by integrating active ageing in urban and community development [7]. According to the World Health Organization’s guidebook, age-friendliness has eight domains: outdoor spaces and buildings, transportation, housing, social participation, respect and social inclusion, civic participation and employment, communication and information, and community support and health services [7]. Thus, age-friendliness is a cutting-cross concept that requires a systematised framework to ensure the efficiency of policymaking.

Given older people’s mobility limitations, age-friendly development should focus on the community environment [8]. However, age-friendly community policy development often encounters various hindrances. Firstly, many other issues compete with age-friendly community development in urban planning for being the policy priority. Policymakers often prefer developing policies that lead to direct economic growth [9]. Secondly, as gentrification and privatisation become the main approaches of urban space development in this neoliberal era, planning policies tend to consider the wealthy and working population who can bring about capital accumulation [10]. The situation leads to institutional ageism, which drives policymaking away from older people’s real needs [11]. Lastly, due to economic austerity, age-friendly community development tends to lack sufficient government funds [12].

In most countries, policies have a significant influence on community development. Thus, an effective and efficient policy framework is essential to realise and evaluate communities’ age-friendliness [13]. Various frameworks have been developed in age-friendly community studies to indicate essential environmental factors and enhance project performance. However, an efficient policy framework to minimise cost and resolve conflicts of interest between different generations in age-friendly community development remains needed. Therefore, this study defines a needed policy framework as having the following three characteristics. (1) It should solve the conflicting expectations between different generations; (2) it should attract investments into age-friendly community projects; and (3) it should reflect the actual demands of older residents by considering their voices.

This study aims to develop a policy framework for age-friendly community development. The policies designed under the guidance of the framework can minimise cost and resolve conflicts of interest between different generations in age-friendly community development. Three detailed research questions are raised to achieve the research aim. (1) What theory is suitable to develop such an efficient policy framework? (2) What type of policy framework can be constructed from the theory identified in the first research question? (3) How can policymakers and designers use the policy framework developed in the second research question to facilitate age-friendly community development? This study adopted a scientometric literature review using a mixed analytical method incorporating qualitative and quantitative analysis to answer the first research question. The literature was retrieved from a keyword search for **age-friendly** AND **community** on Web of Science. The systematic review mapped the theoretical development of age-friendly community studies, revealing that Lefebvre’s theory of *the production of space* plays an important role in age-friendly community studies [14]. To answer the second research question, a policy framework was developed through the lens of the production of space, and the framework indicates an efficient way to structuralise and develop age-friendly community policies. To answer the third research question, some policy recommendations were raised by discussing the developed policy framework. This paper responds to advocates in previous studies by proposing a way to counter financial austerity in age-friendly projects and balance the consideration for the older and younger population in urban development. For practice, the policy framework can inspire age-friendly community policymaking in different regions. This paper will present the methodology, results, and discussions in the following sections based on these intentions.

## 2. Materials and Methods

### 2.1. Scientometrics Method

As a systematic literature review method, scientometrics has different definitions [15,16,17]. However, most definitions highlight **quantitative** as a central attribute of the scientometric method, which is also an advantage over conventional literature review [18,19]. Moreover, with the recent development of scholarly databases (e.g., Web of Science, Scopus, Google Scholar, PubMed), computer techniques, and analysis methods, scientometrics helps this study seize development trends and hotspots of age-friendly community studies by visualisation and computer-aid quantitative analysis [20,21,22,23,24].

This study uses scientometrics because it has three advantages in mapping disciplinary development over the traditional literature review. (1) It can give an objective overview of a research area to inspire further research development [25,26]; (2) it can quantitatively evaluate scientific performance and influence [27]; and (3) it can systematically map a research area’s changing knowledge structure and evolutionary development [28,29].

Scientometrics literature reviews remain rare in age-friendly community research. Until this research, there have been 13 literature reviews in the research area. Ten of these literature reviews are traditional qualitative studies [30,31,32,33,34,35,36,37,38,39], and there are only two emerging scientometric studies on age-friendly communities. Xiang et al.’s systematic review illustrates the evolutionary trend of age-friendly community studies [40]. De Oliveira et al. proposed some suggestions for age-friendly community development in their scientometric research [41]. The current study pays attention to the theoretical aspect of age-friendly community studies because innovative models can be developed by investigating problematic issues through a theoretical lens. The next part will describe the details of the scientometric methods used in this study.

### 2.2. Data Collection and Analysis

We searched Web of Science using the keywords **age-friendly** and **community** to find the previous literature on age-friendly communities. This search strategy was used for two reasons. First, this study regards age-friendly as a distinctive term developed from active ageing containing a comprehensive factor checklist as shown in the World Health Organization’s guidebook [7,42]. Thus, this search only includes the papers exactly quoting the term “age-friendly”. Secondly, the strategy ensured that all the studies on community age-friendliness were included in the search. Sometimes, age-friendly and community separately appeared in the title, abstract, and keyword list, and detailed scrutiny helped identify this type of paper.

**Figure 1:**
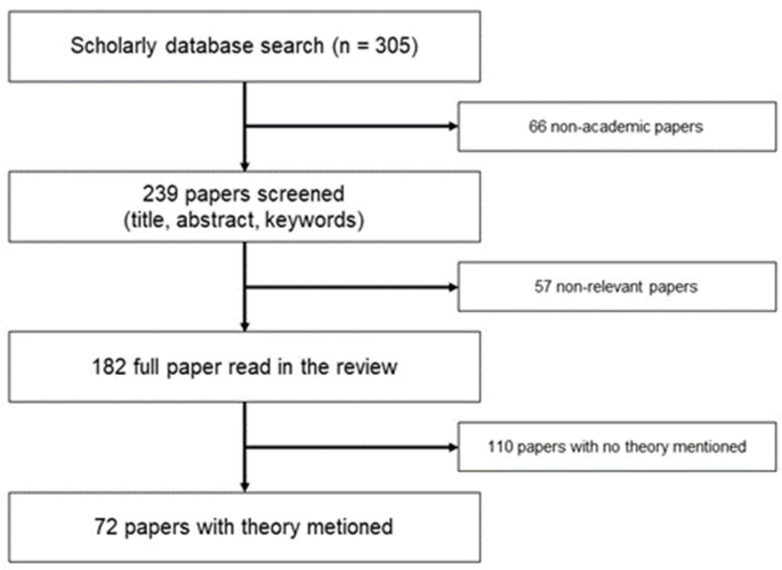
Paper selection through PRISMA’s procedure.

A search in September 2019 returned 305 papers. Preferred Reporting Items for Systematic Reviews and Meta-Analyses (PRISMA) (Figure 1) was used to filter these papers [43]. After scrutinising the title, abstract, and keywords, 182 academic papers on age-friendly communities were left. Finally, after reading all these 182 papers, 72 papers were found containing some explicit theories. Most of these 72 studies were conducted in the Global North, including Canada, the United States, and European countries. Two criteria for literature selection were adapted in the final stage. The first criterion was to review those papers, introducing other new theories into age-friendly community studies. All the 182 academic papers can be regarded as containing some theories because age-friendliness is a theoretical concept itself. Another two theoretical concepts, *active ageing* and *age-in-place*, also appear frequently in these 182 academic papers. However, many innovative frameworks were developed by using other theories, rather than only age-friendly, active ageing and age-in-place. Thus, age-friendly, active ageing, and age-in-place were only regarded as the common terms to show the research topic rather than specific theories in this study. The second selection criterion was that the papers should use the theories in their discussion section.

Using a mixed analytical method, this study mapped the theoretical development of age-friendly community studies and revealed how the 72 identified papers influence the knowledge structure of the research area. The mixed analytical method incorporated qualitative and quantitative parts. The qualitative part summarised the use of each theory with a conventional review method. The theories were further grouped into six categories. The quantitative part evaluated the influence of the 72 papers on the knowledge structure of the research area. A paper’s influence is usually revealed by its citation relationships [44]. Thus, in this paper, publication citations were quantitatively analysed. The citation relationships were processed using two quantitative analysis methods: core publications analysis and publication cluster [45,46]. The algorithms behind these two analysis methods can be found in the related literature [45,46].

Two types of commonly used scientometric software, namely, VOSviewer (Centre for Science and Technology Studies, Leiden University, Rapenburg, The Netherlands) and CiteNetExplorer (Centre for Science and Technology Studies, Leiden University, Rapenburg, The Netherlands), were used in this study. Both types of software can construct citation networks and visualise them. With the network, these two types of software can realise more advanced quantitative analyses to map the science development of a research area. The network produced by VOSviewer is undirected, while the network given by CiteNetExplorer has a temporal direction. They also have different analysis functions. Thus, they supplemented each other in this study.

## 3. Results

### 3.1. Theoretical Trend

The PRISMA identified 182 academic papers on age-friendly communities. However, only 72 papers contain explicit theories. A qualitative analysis of these 72 papers indicates the theoretical trend of age-friendly community studies. This part will summarise the theoretical trend from the perspective of three processes related to age-friendly community development.

The three processes were summarised by reviewing the previous studies and referring to the governance dimension of Lui et al.’s model, which comprises top-down and bottom-up processes [30]. In the top-down process, policymakers’ policies, the community design profession’s masterplans, and community administrators’ regulations shape the environment of communities. This process was named *top-down age-friendly community building* in this study. The studies on top-down age-friendly community building usually identify the environmental factors that can improve community age-friendliness. Some of these studies also explore how to develop the policies and conduct projects for age-friendly communities. In the bottom-up process, older and younger residents use community facilities to get their intended benefits. This process is named *bottom-up age-friendly community using* in this study. The studies of the age-friendly community using mainly focus on the various influences of the community environment on the residents, especially older people. However, conflicts of interest usually exist between the older and younger residents in community building and using. Thus, between the top-down process and the bottom-up process, policymakers, the community design profession, and community administrators need to mediate the conflicts. This process is named *age-friendly community negotiating* in this study, representing the conversations between community planners and community users. The negotiation results can finally influence policies, master plans, and regulations of age-friendly community development. The studies on age-friendly community negotiating usually advocate for involving older residents in community design.

Ecological theory is the most frequently used among all the theories. For **top-down age-friendly community building**, one influential study used five basic assumptions of ecological theory to conceptualise age-friendly communities [42]. The principle of person–environment fit also inspired studies to explore top-down age-friendly community building in different contexts for diverse older people [47,48,49,50,51]. Other studies identified the critical factors in older people’s neighbouring environments to compensate for their vulnerability [52,53,54]. For **bottom-up age-friendly community using**, another influential model, named constructive ageing model, shows six developmental psychological benefits brought by age-friendly communities [55,56]. In addition to psychological wellbeing, studies showed that older residents could also receive better life satisfaction, health, mobility, and participation in age-friendly communities [57,58,59,60,61,62,63,64,65,66,67,68,69,70,71,72].

The production of space is the second most used theory in age-friendly community studies. For **top-down age-friendly community building**, the theory was mainly used to identify the various barriers to age-friendly community development [10,11,73]. Some solutions to the identified barriers were also proposed [9,11]. For **age-friendly community negotiating**, older people’s participatory role in community design was emphasised through the successful examples from the previous projects [74,75,76]. For **bottom-up age-friendly community using**, the spatial use patterns of older residents were frequently studied because daily life is a prominent aspect of the theory [77,78,79].

Other theories were significantly less used in the age-friendly community studies. However, according to the review, the rest of the theories were categorised into four theory groups based on their shared characteristics. These four groups were named social-related theories, place-related theories, governance-related theories, and individual-centred theories.

Some studies investigated age-friendly communities from their social aspect. For **top-down age-friendly community building**, social connectivity was identified as an essential factor to facilitate age-friendly community projects (social connectivity: [80]). Some theories were also used to explore the ways to create a respectful community environment (social capital [81] and research-based art [82]). However, caution was also raised that some inappropriate measures may strengthen the negative images of ageing in age-friendly communities (social constructivist: [83]). For **age-friendly community negotiating**, the different perceptions of communities were found between developers and older residents (social constructivist [84,85]). Thus, social resources and practices were both advocated to facilitate older residents’ engagement in community design (social capital [86], social connectivity [87], cultural capital [88], co-production [89], intergenerational practice [90], and co-design: [91]). For **bottom-up age-friendly community using**, age-friendliness was found to improve older residents’ wellbeing and mitigate some negative factors in the community environment (health equality [92], studentification [93], broken window [94], social exchange [95], social production function [96], and social integration [97]). Age-friendly cities and communities can turn their older citizens into active contributors to sustainable urban development (SUD). Han et al. suggest a strategy for policymakers, especially at the municipal level, to incorporate the concept of age-friendly communities in SUD [98]. However, the ignorance of local context and individual heterogeneity may exacerbate the deprived situation of some vulnerable residents in age-friendly communities (spatial inequality: [99], postcolonial theory: [100]).

Another main theoretical development trend of age-friendly community studies is to use place-related theories. For **top-down age-friendly community building**, community characteristics were suggested to be carefully considered in community design, especially to identify the communities with a higher probability to encounter natural disasters (hazard of place [101] and place integration [102]). For **bottom-up age-friendly community using**, age-friendly communities were found to retain older residents in their original living place and enhance their mobility (image of the city [103] and place attachment [104]).

Governance is another essential issue in age-friendly community studies [30]. Thus, governance-related theories are also frequently used. For **top-down age-friendly community building**, this type of theory was used to investigate the effectiveness and efficiency of specific policymaking and projects (community change [105], natural and neutral organisational characteristics [106], sustainability [107], local governance [108], power distance [109], knowledge production [110], and agenda-setting [111]). Some studies also analysed the influence of policy text on top-down age-friendly community building practice (post-structural theory [112] and the logic of choice [113]).

Recently, a trend has emerged in which some individual-centred theories are used in age-friendly community studies. For **bottom-up age-friendly community using**, the research outcome suggested that age-friendly communities can enhance the social and psychological development of older residents (identity theory [114] and Erikson psychological development model [115]). The emerging trend opens a new research perspective questioning the traditional age-friendly community development mode. As active and successful ageing is the underpinning concept of the age-friendly community, these studies also urge the policymakers to rethink whether they should produce a place to provide various conventional care services or a place to increase the active level of older residents and make them capable of taking good care of themselves.

The qualitative analysis clusters 72 papers into six theory groups. The *ecological theory* is the most used in previous age-friendly community studies. It was used to study top-down age-friendly community building and using. *The production of space* is the second-most used theory and was used to study all three processes. *Social-related theories* were also used to study all three processes. *Place-related theories* were used to study age-community building and using. *Governance-related theories* were used to study top-down age-friendly community building. Lastly, *individual-centred theories* were used to study the bottom-up age-friendly community using. The following parts of this section will focus on the quantitative analysis of the influence of the six theory groups.

### 3.2. Longitudinal Analysis of the Theoretical Trend

The longitudinal trend of using each theory group in age-friendly community studies is shown in Table 1. This trend has two significant characteristics. Firstly, the used theories have become diverse. Until 2013, only one or two types of theories appeared in age-friendly community studies each year. After 2014, the theories used to study age-friendly communities were diverse each year, except in 2017. Secondly, the number of identified papers was increasing continuously. After 2014, a significant increase was found in the publications containing some explicit theories. In 2018 and 2019, the number of identified papers exceeded 10 in each year. The longitudinal trend indicates the importance of introducing new theories to develop the research area in recent years.

### 3.3. Scientometrics Analysis of Theoretical Trend

To explore the influence of the identified theories on the knowledge structure of age-friendly community studies, all the 182 academic papers were analysed quantitatively to determine if any of the 72 theoretical publications are core or central publications. Using the **core publication analysis** of CitNetExplorer can help identify the core publications in a citation network. According to the definition given by CitNetExplorer, core publications are the papers that have more than a certain number of citation linkages with other core publications in the citation network, and the specific number is a parameter usually set by researchers according to their intention and experience. The counted citation linkages have no directions in the analysis, meaning that a core publication is either citing or cited by other core publications. Thus, the influence of core citations was defined as integrating and disseminating knowledge in this study. This study also used the **publication cluster** of VOSviewer to cluster the 182 papers into several groups according to their citation relationships. The groups are formed based on the closeness between publications through direct or indirect citation linkage. Citation linkages analysed in the publication cluster also have no directions. Thus, the influence of publications is similarly defined as integrating and disseminating knowledge. The clustering makes the knowledge structure of the research area clearer, and influence impacts within a cluster are more potent than those between different clusters. A central publication was identified in each cluster in this study. The definition of a central publication is that the paper has the most citation linkage in the whole citation network of each cluster. Thus, a central publication can be influential within and outside its cluster.

**Figure 2:**
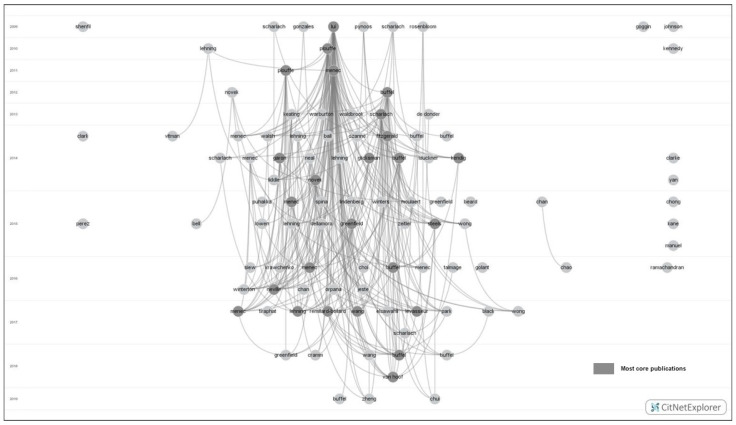
Most-core publications among age-friendly community studies (K-core algorithm: K = 7).

**Figure 3:**
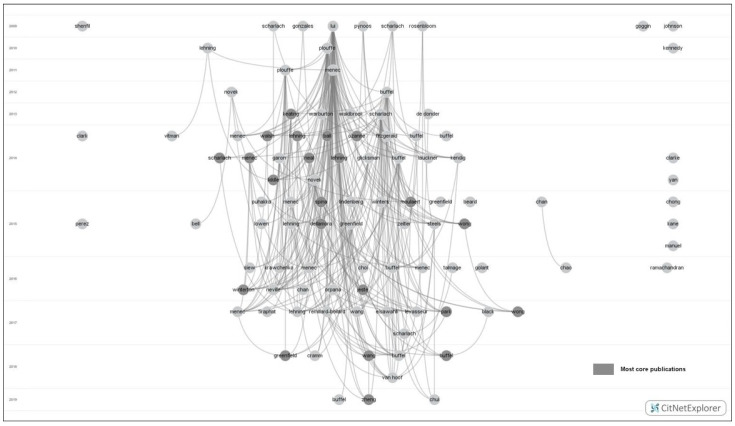
More-core publications among age-friendly community studies (K-core algorithm: K = 5 and 6).

Using the core publications analysis, the 182 papers were categorised into four groups: *most-core publications* (Figure 2), *more-core publications* (Figure 3), *less-core publications,* and *non-core publications*. Given that the parameter of core publication analysis is set by researchers’ intention and experience, this study assigned different parameters to different types of core publications. When setting k as 8, no core publications were identified. Thus, when setting k as seven, the identified core publications were regarded as most-core publications. The most-core publication means those publications with seven citation linkages with other most-core publications. When setting k as five and six, the additionally identified core publications were more-core publications. The more-core publication means those publications with five or six citation linkages with other more-core or most-core publications. When set k below five and above zero (excluding five and zero), the additionally identified core publications were less-core publications. The less-core publication means those publications with less than five citation linkages with other core publications. Non-core publications were those publications that cannot be identified by core publication analysis. The non-core publication means those publications with no citation linkages with other core publications. Table 2 shows the detailed result of the core publications analysis.

A total of 63 out of the 72 theoretical studies were identified as core publications. Table 3 is a cross table of the types of core publications and theory groups. Three theory groups contain most-core publications: ecological theory, production of space, and social-related theories. Thus, these groups of theories can explain a wide range of issues in age-friendly communities by either citing or being cited by other publications. Some common research topics are also identified in these most influential publications, including **contextual and individual differences** [51,92], **social connectivity** [42,80,81,87], **participatory design** [74,87], and **barriers and facilitators of age-friendly communities** [9,10].

**Figure 4:**
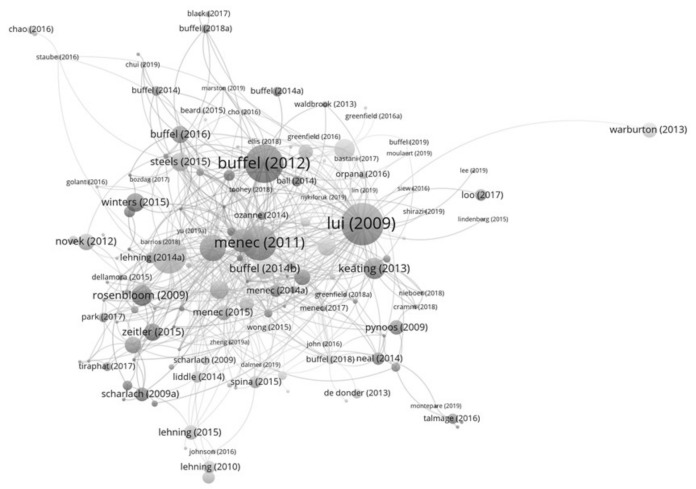
Citation analysis of age-friendly community studies.

Using VOSviewer, the citation map gave more information about the theoretical development trend of age-friendly community studies. As illustrated by the visualised citation map (Figure 4), among the 182 papers, Lui et al.’s [30] literature review remains the most influential. The second most influential publication is Buffel et al.’s [74] work belonging to the production of a space group. The third most influential is Menec et al.’s [42] paper with an ecological perspective. The citation map was clustered into 18 groups by the publication cluster function of VOSviewer. However, some groups only contain a minimal number of publications. Thus, this study focused on clusters with more than 10 papers, and seven larger clusters were identified and further analysed. According to the aforementioned definition of the central publication, seven central publications were identified from the analysed clusters. Table 4 shows the details of these seven publication groups. Among these seven central publications, five of them are theoretical publications. The social capital theory was used in the central publication of Cluster One, which is the largest cluster. The central publications of Clusters Three and Five are from the ecological theory group. The central publications of Clusters Four and Seven are from the production of space group. Again, the citation map produced by VOSviewer shows that ecological theory, the production of space, and social-related theories, which are the three types of influential theories in age-friendly community studies.

## 4. Discussion

### 4.1. Theory Comparison and Selection for Framework Development

This part will explore the theoretical development of age-friendly community studies from two aspects, theory capability and theory influence. Firstly, the theory capability will be studied by referring to an age-friendly community life cycle. Then, the theory influence will be evaluated by discussing the quantitative results of the scientometric analysis.

**Figure 5:**
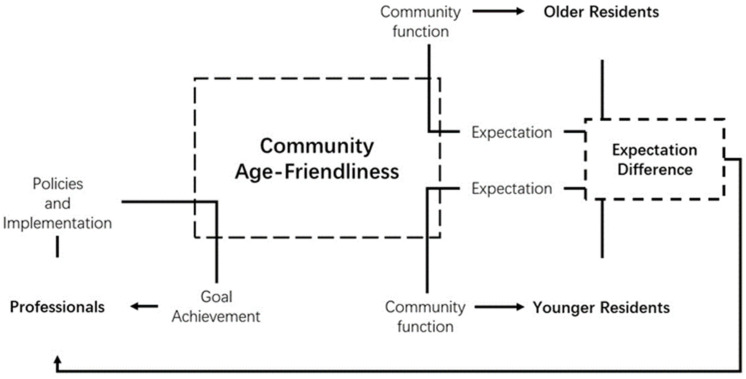
Life-cycle of community age-friendliness.

These three processes related to age-friendly community development can form an age-friendly community life cycle. Although the World Health Organization has developed a life cycle of age-friendly initiatives for Global Network for Age-friendly Cities and Communities members [11], this official life cycle is mainly applied to project management, and it lacks a deeper insight into the residents’ life experiences. Thus, the life cycle model in this research fulfils the gap to serve the purpose of this study. The model is visualised in Figure 5. It helps evaluate the capability of each group of theories by exploring (1) whether the theory or theories can be used to analyse a particular stage of the life cycle and (2) the strengths and drawbacks when using the theory or theories. Overall, the model has three stages: top-down age-friendly community building, age-friendly community negotiating, and bottom-up age-friendly community using. A detailed explanation of the three stages can be retrieved from this paper’s related text.

The ecological theory takes a person–environment fit perspective, in which the environment determines people’s behaviour and wellbeing. The theory was used to study top-down age-friendly community building and using. However, taking an environmentalism perspective means that older people are regarded as passive users of their environment in the theory. This characteristic of the theory may be why it is seldom used to study age-friendly negotiating. The production of space studies the interaction between the form of space and the social relations within it. Although the theory was used to study the whole age-friendly community life cycle, to the best of the authors’ knowledge, it was seldom used to develop a framework to guide age-friendly community development. This gap will be filled in the latter part of this paper. The social-related theories are more capable of solving the issues in the community social environment. These theories were used to study the whole age-friendly community life cycle. In comparison, place-related theories focus more on the community physical environment. These theories were mainly used to study top-down age-friendly community building and using. Given that age-friendly community environments contain social and physical aspects, social-related theories and place-related theories need to supplement each other to depict a full view of the age-friendly community. Governance-related theories were mainly introduced from management science and politics to study the specific policymaking and projects. The theories were used to analyse top-down age-friendly community building from a top-down perspective. By contrast, individual-centred theories are mainly introduced from psychology to study bottom-up age-friendly community using from a community users’ perspective. Governance-related and individual-centred theories also need to supplement each other to analyse age-friendly community governance, which contains top-down and bottom-up processes.

Overall, three gaps were found in the previously used theories in age-friendly community studies. (1) The ecological theory regards older people as passive users of community space and neglects the active role of older residents in age-friendly community negotiating. (2) Social-related and place-related theories lack physical and social perspectives, respectively, in studying age-friendly community environments. (3) Governance-related and individual-centred theories lack bottom-up and top-down perspectives respectively in studying age-friendly community governance.

The production of space can address these three gaps. According to the theory, residents are active actors in shaping a community by giving meaning to community spaces. Regarding the second limitation, the theory can integrate the physical and social aspects by investigating how social relations and spatial form interact. Lastly, the theory also focuses on the interactions between spatial design policies and the spatial meaning given by residents. A detailed explanation of the theory will be given in later. As mentioned, to the best of the authors’ knowledge, the production of space has not been used to develop any theoretical framework in age-friendly community studies. This study will attempt to fill this gap by developing a policy framework using the theory.

The results of the scientometric analysis show the influence of each group of theories. The diversity of the theories used in the age-friendly community study has been increasing with time. The social-related theory group has had the most extensive expansion these years, which indicates an emerging interest in the social environment of the age-friendly community. According to core publication analysis and publication cluster, ecological theory, and the production of space and social-related theories are the three influential theory groups in the knowledge structure of the age-friendly community study. The analysis results reflect the research interests in person–environment interaction, community building through social relations, and the social environment in age-friendly community studies. The identified influential research topics indicate how research should help solve the barriers currently faced by age-friendly communities. One main barrier is that communities are mainly designed for the working population because local governors prefer regional economic growth. Participatory community design is advocated to incorporate individuals’ living experiences in community design. Social connectivity can play an important role in facilitating the evolvement of older residents in community design decisions. As shown in previous studies, social connectivity means both the relationship between older people and their neighbours and a broad collaboration between various stakeholders of age-friendly communities.

The production of space has advantages in dealing with these influential research topics. (1) The barriers to age-friendly communities are identified through a lens of the production of space. Thus, giving the solutions using the same theory is suitable. (2) The theory focuses on studying users’ daily experience in community spaces by exploring their spatial using patterns and the meanings they give to community spaces. (3) The theory is centrally concerned with investigating conflicts and compromises between spatial design policies and the meanings given by spatial users. This concern is also the nature of the participatory design. (4) The basic principle of the theory is to reveal the relationship between social relations and spatial form. Thus, in the next part, the theory of the production of space and its potential contribution to age-friendly community study will be used to develop a policy framework for age-friendly community development.

### 4.2. Policy Framework Development Based on the Production of Space

The production of space is a theory about social space [14]. The core discourse is the *socio-spatial dialectic*: Social relations shape the space, while space reinforces the social relations [78]. The theory realises the analysis of social order from the perspective of the spatial form [116]. It also provides a method to control the physical form of space using social relations. Lefebvre used a spatial triad containing spatial practice, spatial representations, and representational spaces to analyse the production of space [14,117,118]. In the production of space, every stakeholder is actively shaping the space. In the top-down process, community design professions and governors shape the space using the representation of space, which is the professional knowledge about the space. The spatial practice of these hegemonic actors is either commodification to achieve exchange values or bureaucratisation to enhance administration [10,119]. Through these practices, the abstract space of bourgeoisie and capitalism is shaped into the lived space of public users. In the bottom-up process, space has use-value for its residents. Residents live in a representational space, which is the symbolic space of the meanings given by the residents [73]. Representational spaces determine the user’s behaviour in the space, which is the users’ spatial practice [79].

Different user groups may usually give the same space conflicting meanings [120]. In commodification and bureaucratisation of space, hegemonic actors need to decide which user group is their priority [9]. Thus, having its root in Marxism, the theory reveals that urban space is built on the conflicts between social groups [75]. These intergroup conflicts can lead to spatial practice conflicts between different user groups [77]. In the worst situation, segregation and inequality may even be generated from spatial conflicts [10,77]. In the production of space, such segregation and inequality are a loss of the right to space, which means the ability to shape and use the space [74,90]. Negotiation is essential to mediate the conflicts between representational spaces and spatial representations [121]. In practice, the negotiation usually takes the form of participatory design. However, deprived social groups may lose their access to the negotiation because of losing the right to space [122]. There are also some examples found from the empirical studies. In Australia, Jenkin et al. found that older people are treated as a less attractive customer segment by some community organisations, as an important stakeholder of policymaking [123]. Rémillard-Boilard et al. also suggest policymakers should change their mindset to see older people according to their study in 11 countries worldwide [124]. The low priority of older people’s issues in policymaking is also found in empirical studies. In China, Li and Woolrych found that older people’s issues tend to be excluded from smart city development [125]. While in Poland, Podgórniak-Krzykacz et al. found that both smart cities and age-friendly communities are of low policy priority [126]. In Canada, Joy finds that the neoliberalism policy atmosphere has a significant impact on the policymaking for older people [127]. In the UK, Murtagh et al. found that property economy may cause the risk to deprive older people’s rights in policymaking [128]. Ignorance of the heterogeneity within the older age group is also the main policymaking problem recorded in many real cases. In the UK, Yazdanpanahi and Hussein found that policies for older people usually take a generic and homogeneous lens and neglect the rights of some minority ethnic groups [129].

At present, age-friendly community policies are still being developed slowly because the hegemonic actors of community spaces are reluctant to put age-friendliness factors in their spatial representations [130,131]. One of the main reasons for this reluctance can be attributed to institutional ageism in policymaking [132]. Using the production of space, Buffel and Phillipson analysed the barriers confronted by age-friendly community policy development [9]. The most fundamental barrier is the intergenerational inequality to determine urban development. Affected by neoliberalism, urban development mainly serves capital accumulation and economic development. Thus, community spaces mainly serve the younger working population and the participants of urban economic activities. However, the younger generation usually has conflicts with older residents over spatial expectations and spatial practices [133]. This institutional ageism in policy development reflects the wide societal ageism that discriminates older people because of the stereotype of an economic burden [134].

Developers and administrators also tend to resist improving the age-friendliness of the communities due to the high costs, low profits, and adverse effects on the communities’ revenue and economic development [130]. The universal design and specialised facilities for older residents in communities are usually considered extra community development costs [135]. Furthermore, ensuring a suitable environment for older people can increase energy consumption mainly because of elevator dependency, lighting, temperature control, and some 24-h services [136]. Age-friendly community costs also include paying for the workforce in the essential services for older residents. The work in age-friendly communities usually requires more devotion and skills than ordinary service jobs. Thus, a significant expenditure needs to be incurred on workers’ salaries and training and development [137]. Moreover, qualified labour to serve older residents remains limited [138]. Lastly, age-friendliness is usually related to the welfare provision in communities. The fact that that these facilities and services should be at a low price, considering older people’s economic conditions, is taken for granted, leading to the low profits of age-friendly facilities and services in the community [139]. Given the higher profits commercial services bring, community spaces tend to be allocated to commercial utilities.

Under such circumstances, older residents tend to lose their right to community spaces. Some countries regard older people’s right to community spaces as a human right and put the right in law [140,141]. However, the enforcement of the laws is usually tricky due to the low priority of older people’s rights in the national law system [142]. Thus, older residents may lose their access to some community spaces for younger residents’ convenience. Like many other social groups that lose their human rights, older residents may also lack the opportunities to express their perception of community spaces to the hegemonic actors in community building. As a result, the spatial segregation of older residents is prevalent in the community. Older residents can only stay lonely at home or outdoors without any active social or civic participation.

Thus, from the perspective of the production of space, a strategy dimension of the policy framework is formed. This dimension can facilitate age-friendly community policy development in the following three ways. Firstly, the framework should commodify age-friendly communities. It should boost the profits of age-friendly communities and involve the various stakeholders who can produce the profit. Thus, age-friendly communities can become a method of capital accumulation of the city. Second, the framework should institutionalise age-friendly communities. It should redistribute community spatial resources to eliminate the inequality between different generations and reduce the ageism in community administration. By bureaucratisation, the necessary community spaces for older people to use can be ensured. The regulated use of space also means the protection of older residents’ rights to community spaces. Lastly, the framework should increase the opportunities for older residents to participate in the negotiation of community space building. By expanding their social connectivity, older residents can participate in age-friendly community negotiating directly or indirectly through their social network in communities.

**Figure 6:**
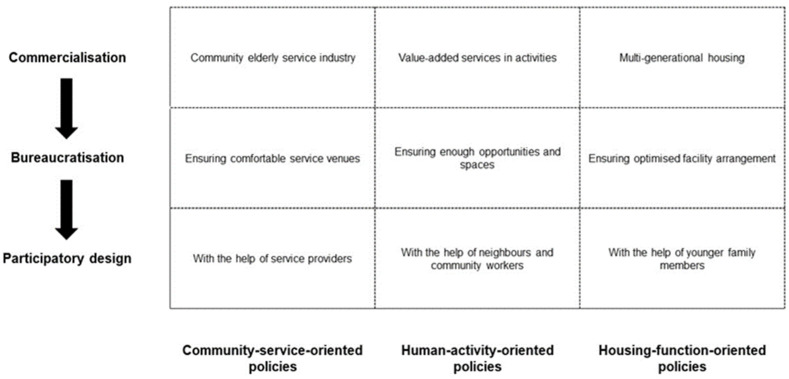
Policy framework for age-friendly community development.

Some communities intentionally built for older people, such as continuing care retirement communities, give an ideal model of age-friendly communities. In these communities, the main purchasing power is older residents. Thus, the profits are derived from older residents. The administration is also designed specifically for older residents’ daily life. The representations of space in these communities consider their older residents mainly from three aspects: community services, human activities, and housing functions. Thus, this paper develops such a policy framework (Figure 6), including three sets of policies: community-service-oriented, human-activity-oriented and housing-function-oriented. These sets form the component dimension of the policy framework. However, it is difficult to only consider the spatial expectations of older residents in ordinary urban communities because of a more complex resident demographic structure. Thus, in the policy framework, the strategy and component dimensions should interact with each other. The following part will explore the policy implication of the framework in detail by discussing the interaction between the framework’s component dimension and strategic dimension.

### 4.3. Implications of the Framework in Policymaking

Community-service-oriented policies regulate essential services for older people in communities. To commodify age-friendly communities, this type of policy can encourage service providers to conduct business in communities. This measure can promote the elderly service industry within communities and trigger regional economic activities. Thus, older residents can be regarded as “real” customers and be considered more in community building. By bureaucratising community services, service providers can have convenient access to suitable venues for elderly services in communities. Although communities usually tend to leave enough space for daily services, the physical conditions of these venues may not satisfy the requirements of the services for older people [143]. With bureaucratisation, service providers can coordinate with community designers to ensure the spaces are comfortable for older residents to access and stay. Lastly, community services can expand the social support network of older residents. Older residents can have sufficient contact with service providers, and their spatial demands can also be expressed with the help of the service providers.

Human-activity-oriented policies ensure that older residents have sufficient opportunities to participate in community activities. In commodification of the age-friendly community, the policies can introduce commercial factors into community activities. These commercial factors can be the value-added services of the activities. The bureaucratisation of community activities officially ensures enough opportunities and community spaces for carrying out activities. Such formalisation of older residents’ activities can also decrease the conflicts of the spatial practice between older residents and their younger neighbours. Lastly, the regulated activities expand older residents’ social network with their neighbours in the community, including the older neighbours, younger neighbours, and community workers. These activities can encourage intergenerational interaction in the community and increase the younger generation’s awareness of their neighbouring older residents. Thus, older residents’ community spatial expectations can be expressed by their younger neighbours and community workers.

Housing-function-oriented policies focus on housing in communities by incorporating multi-generational design principles. Housing is a topic of age-friendly communities covering many issues from interior design to surrounding facilities [144]. At present, with the preference for multi-generational housing, living with their parents to take care of them becomes the main reason for the younger generation to live with or nearby older residents [145]. Thus, policies should concentrate on the common benefit of older and younger generations in housing development. In commodifying age-friendly communities, policies should encourage the supply and demand sides of multi-generational housing. Thus, the facility and housing design should facilitate younger people to balance their jobs, daily life, and caregiving to their parents. These facilities and housing can become the value-added components to enhance the exchange value of communities. The bureaucratisation of intergenerational infrastructures and multi-generational housing ensures spatial equality between generations by using environmental arrangement strategies, regulations, and techniques to redistribute community resources. Thus, the spatial resource in the community should be optimised to ensure the efficiency demanded by the working population and the accessibility when older residents demand the facilities. Lastly, older residents maintain the closest social support relationships in their communities with their children living with them. Thus, older residents’ spatial demands can be expressed by their children.

Overall, guided by the theory of the production of space, each set of policies can be divided into three sections. (1) Guided by *space is built for capital accumulation* [146], commodification should be the basic principle. It encourages the related commercial stakeholders to participate in age-friendly community development. With proper commercialisation, age-friendly communities can become a new capital exchanging market in cities, especially with the rapidly expanding aged population. This transformation can help age-friendly communities earn more support from policymakers because of its economic contribution. (2) Guided by *different groups’ right to space should be ensured* [147], the bureaucratisation of age-friendly communities redistributes community spatial resources and ensures older residents can have the right to community spaces. It should regulate the management and use of community spaces. Such formalisation of older residents’ spatial practice can also reduce the intergenerational conflicts over community space using. (3) Guided by the *socio-spatial dialectic* [78], the policy can realise older residents’ participatory planning in top-down age-friendly community building by directly involving older people or indirect involvement through their social relations. Through the commercialisation and bureaucratisation of age-friendly communities, older people can establish solid relationships with their family, neighbours, and other related stakeholders in their communities. These relationships can enhance the negotiation power of older residents in the production of community spaces. These policies’ details can be designed by localising the WHO’s checklist for age-friendly cities and communities [7]. The detailed localisation methods need more future research on this policy framework.

## 5. Conclusions

### 5.1. Research Conclusions

Owing to a lack of policy framework to facilitate efficient age-friendly community policy development in the face of various barriers brought by neoliberalism, this article makes an effort to develop such a framework to minimise cost and resolve the conflicts of interest between different generations in age-friendly community development. As a result of a systematic literature review, the theory of the production of space was found to be influential in age-friendly community studies and capable of solving the problematic issues faced by age-friendly community policymaking. However, the theory is seldom used to develop theoretical frameworks in the research area. Thus, this study’s significant contribution is creating a policy framework based on the production of space for studying age-friendly community development (Figure 6). The policy framework contains community-service-oriented policies, human-activity-oriented policies, and housing-function-oriented policies. In detail, each set of policies should have three parts. (1) It should enhance proper commercialisation through inviting related commercial stakeholders to make age-friendly communities profitable for urban economic development; (2) it should provide some administrative regulations in age-friendly communities to ensure spatial equality between older and younger residents; and (3) it should encourage older residents’ participatory planning either directly or indirectly through their community social network.

### 5.2. Research Limitation and Further Study

However, this study is merely a theoretical development of the policy framework. The theme of this research is still not specified enough for the practice in the real world. Thus, the future development of this policy framework can be better realised through three-phase empirical studies. In the first stage, the current age-friendly community policies should be reviewed to evaluate how well they reflect this policy framework and find the evidence for its effectiveness. Then, more empirical studies should be carried out to evaluate the effects of this policy framework on different stakeholders. Lastly, researchers can bring the mature theoretical framework to policymaking practice by developing more practical guides.

It is recommended that this kind of systematic review be continuously conducted as more studies emerge in the research field. After September 2019, there were 156 new papers in the research field, and 42 of them were theoretical studies. The new papers can still reflect the theoretical trend found in the analysis of this study. It validated that scholars still use the six groups of theories to study age-friendly community: (1) **ecological theory** [148,149,150,151,152,153,154,155], (2) **the production of space** [156,157,158,159,160,161], (3) social-related theories [98,162,163,164,165,166,167,168,169], (4) **place-related theories** [170,171,172,173,174,175,176], (5) **governing-related theory** [177,178,179,180,181], and (6) **individual-centred theories** [182,183,184,185,186,187,188]. In addition, more researchers find the importance of studying the social dynamic and individual experience in age-friendly community development. However, after 2020, COVID-19 has had a relatively significant influence on the research topic of age-friendly community studies. Thus, it is also necessary to study the literature after 2020 separately to explore the most cutting-edge research trends in the pandemic and post-pandemic era.

This study may also trigger some more interesting research thoughts for further exploration. For example, some readers may be interested in the influence of cultural differences on the political barriers to age-friendly development in various regions. The question could be solved with an international comparative empirical policy study, as it needs to evaluate the culture and characteristics of the political disputes systematically. As social-related and individual-centred theory is becoming significant in the research field, it is also interesting to see whether policymakers have the same concern in their practice in future studies.

## Figures and Tables

**Table 1:** Publication trend of different theory groups in 2009–2019.

Theory	Publication Number (Percentage within Each Year)										
	2009	2010	2011	2012	2013	2014	2015	2016	2017	2018	2019
Ecological	1 (11.11%)	-	1 (50.00%)	-	1 (20.00%)	3 (13.04%)	4 (19.05%)	6 (24.00%)	3 (12.50%)	5 (17.86%)	3 (7.50%)
Production of space	-	-	-	1 (50.00%)	-	1 (4.35%)	2 (9.52%)	2 (8.00%)	-	1 (3.57%)	3 (7.50%)
Social-related theories	-	-	-	-	2 (40.00%)	2 (8.70%)	1 (4.76%)	1 (4.00%)	5 (20.83%)	6 (21.43%)	3 (7.50%)
Place-related theories	-	-	-	-	-	1 (4.35%)	2 (9.52%)	-	-	-	1 (2.50%)
Governance-related theories	1 (11.11%)	-	-	-	-	1 (4.35%)	1 (4.76%)	3 (12.00%)	-	2 (7.14%)	1 (2.50%)
Individual-centred theories	-	-	-	-	-	-	-	-	-	-	2 (5.00%)
**Total per year**	2 (22.22%)	0 (0.00%)	1 (50.00%)	1 (50.00%)	3 (60.00%)	8 (34.78%)	10 (47.62%)	12 (48.00%)	8 (33.33%)	14 (50.00%)	13 (32.50%)

**Table 2:** Number of publications in different core publication categories.

Category	K	Identified Publications	Net Value
Non-Core Publications	0 (assumed)	182	29
Less-Core Publications	1	153	28
	2	125	18
	3	107	22
	4	85	25
More-Core Publications	5	60	14
	6	46	16
Most-Core Publications	7	30	30

**Table 3:** Number of each type of core publication in each theoretical group.

Theory	Publication Number (Percentage within Each Core Category)			
	Most-Core	More-Core	Less-Core	Non-Core
Ecological theory	2 (6.67%)	6 (20.00%)	16 (17.20%)	3 (10.34%)
Production of space	3 (10.00%)	0 (0.00%)	6 (6.45%)	1 (3.45%)
Social-related theories	4 (13.33%)	2 (6.67%)	12 (12.90%)	2 (6.90%)
Place-related theories	0 (0.00%)	1 (3.33%)	1 (1.08%)	2 (6.90%)
Governance-related theories	0 (0.00%)	3 (10.00%)	6 (6.45%)	0 (0.00%)
Individual-centred theories	0 (0.00%)	1 (3.33%)	0 (0.00%)	1 (3.45%)
**Total of each core category**	9 (30.00%)	13 (43.33%)	41 (44.09%)	9 (31.03%)

**Table 4:** Seven largest publication clusters of age-friendly community studies.

Cluster	Size	Central Publication	Theory Group of the Central Publication
1	19	Scharlach and Lehning, 2013	Social-related
2	14	Rosenbloom, 2009	-
3	14	Keating, Eales and Phillips, 2013	Ecological
4	11	Buffel, Phillipson and Scharf, 2012	Production of Space
5	11	Novek and Menec, 2014	Ecological
6	10	Pynoos, Caraviello and Cicero, 2009	-
7	10	Buffel and Phillipson, 2016	Production of Space

## Data Availability

Not applicable.
